# Chronic exposure to outdoor air pollution and diagnosed cardiovascular disease: meta-analysis of three large cross-sectional surveys

**DOI:** 10.1186/1476-069X-8-30

**Published:** 2009-07-13

**Authors:** Lindsay JL Forbes, Minal D Patel, Alicja R Rudnicka, Derek G Cook, Tony Bush, John R Stedman, Peter H Whincup, David P Strachan, HR Anderson

**Affiliations:** 1 Division of Community Health Sciences, St George's, University of London, Cranmer Terrace, London SW17 0RE, UK; 2 AEA Energy & Environment, The Gemini Building, Fermi Avenue, Harwell, Didcot, Oxfordshire OX11 0QR, UK

## Abstract

**Background:**

Higher exposure to outdoor air pollution is associated with increased cardiopulmonary deaths, but there is limited evidence about the association between outdoor air pollution and diagnosed cardiovascular disease. Our study aimed to estimate the size of the association between long term exposure to outdoor air pollution and prevalent cardiovascular disease.

**Methods:**

We carried out a cross-sectional analysis of data on more than 19,000 white adults aged 45 and older who participated in three representative surveys of the English population in 1994, 1998 and 2003, examining the relationship between self-reported doctor-diagnosed cardiovascular disease and exposure to outdoor air pollutants using multilevel regression techniques and meta-analysis.

**Results:**

The combined estimates suggested that an increase of 1 μg m^-3 ^in concentration of particulate matter less than 10 microns in diameter was associated with an increase of 2.9% (95% CI -0.6% to 6.5%) in prevalence of cardiovascular disease in men, and an increase of 1.6% (95%CI -2.1% to 5.5%) in women. The year-specific analyses showed strongly positive associations in 2003 between odds of cardiovascular disease in both men and women and exposure to particulate matter but not in 1994 or 1998. We found no consistent associations between exposure to gaseous air pollutants and doctor-diagnosed cardiovascular disease.

**Conclusion:**

The associations of prevalent cardiovascular disease with concentration of particulate matter less than 10 microns in diameter, while only weakly positive, were consistent with the effects reported in cohort studies. The results provide evidence of the size of the association between particulate air pollution and the prevalence of cardiovascular disease but no evidence for an association with gaseous pollutants. We found strongly positive associations between particulate matter and cardiovascular disease in 2003 only, which highlights the importance of replicating findings in more than one population.

## Background

Higher outdoor particulate concentration is associated with increased cardiopulmonary deaths in cohort studies[1,2]. Many studies have shown short-term associations between air pollution levels and cardiovascular events up to a few weeks later [3]. However, we have found no reports of large scale population studies of chronic exposure to air pollution and cardiovascular morbidity rather than mortality, except for a cohort study in women which suggested that incidence of cardiovascular disease in women is increased by exposure to fine particulates [4].

To explore the association between chronic exposure to air pollution and cardiovascular morbidity, we examined whether exposure to outdoor air pollutant levels (particulate matter less than 10 microns in diameter (PM_10_), nitrogen dioxide (NO_2_), sulphur dioxide (SO_2_) and ozone (O_3_)) was related to the prevalence of self-reported doctor-diagnosed cardiovascular disease collected during three cross-sectional surveys of the English population carried out in 1994, 1998 and 2003.

## Methods

### Participants and outcome measures

The Health Survey for England is a programme of annual surveys of representative samples of people living in private households in England. We used data on adults participating in 1994, 1998 and 2003, years concentrating on cardiovascular disease and its risk factors.

In each survey year, adults were recruited using a multistage sampling strategy designed to generate a representative sample of the English population [5,6]. Briefly, the sampling units were single postcode sectors (each containing around 2500 addresses; median area about 4 square kilometres (km^2^)), except where the population was very sparse, where they consisted of two or more neighbouring sectors. For each survey year, 720 sampling units, representing about 10% of the total, were selected with a probability proportional to the number of addresses within it. From each selected sampling unit, 19 addresses were drawn (18 in 1994). All households within those addresses were invited to take part, unless they were businesses, institutions or vacant. All adults (up to a maximum of ten) living in each household at those addresses were invited to complete a health questionnaire. We considered people to have cardiovascular disease if they said that they had had a heart attack, angina or stroke that had been diagnosed by a doctor.

### Exposures

We have described methods for assigning exposure in our previous publication [7]. Briefly, the National Centre for Social Research provided postcode sector of residence for all households in the Health Surveys for England in 1994, 1998 and 2003. We assumed that annual average pollutant exposure for people living in each postcode sector was that of the 1 km^2 ^where its centroid was located. We estimated annual average background exposure to PM_10_, NO_2_, SO_2 _and O_3 _for each 1 km^2 ^of England using air dispersion models using meteorological parameters and an emission inventory [8]. Additional file 1 provides further information.

Because data collection for each Health Survey is carried out over the whole year and part of the following year, we averaged the exposure estimate for each participant for each pollutant for the data collection year and the previous year, although, as we did not have pollution data for 1993, for 1994 we used annual averages for 1994 only.

### Statistical analysis

The median number of adults taking part in the Health Survey for England living in each postcode sector (the level at which pollutant exposure was estimated) was 22 in 1994, 21 in 1998 and 21 in 2003. Therefore, we used multilevel logistic regression analyses to take account of the hierarchical structure of the data with a level for postcode sector. We analysed data on white adults aged 45 years or older separately for men and women, to take account of possible clustering of cardiovascular disease within households, but did not allow for household level in the analysis, because there were few households with more than one adult aged 45 and older of each sex.

We examined crude associations within each survey year between each pollutant and risk of self-reported doctor-diagnosed cardiovascular disease using multilevel logistic regression analysis with two levels: postcode sectors formed the upper level and individuals within postcode sector formed the lower level. A random intercept model was used allowing for log odds of the outcome to vary by postcode sector. We then controlled for the following individual level potential confounders: age (10-year age groups); body mass index (quartiles); social class of head of household (UK Registrar General's classification); cigarette smoking (never, ex-, current); and region of residence (8 regions).

We carried out fixed effects meta-analysis of the year-specific estimates for each air pollutant using inverse variance weighting. All analyses were performed using STATA version 9.2 (STATA Corporation, Texas, USA).

### Ethical approval

In 1994, 1998 and 2003, ethical approval was obtained from all relevant Local and Multi-Centre Research Ethics Committees by the National Centre for Social Research. Participants provided explicit consent to take part in the survey. We obtained approval to link air pollution data to Health Survey for England data from the National Centre for Social Research, having undertaken to follow procedures to protect participants' identities.

## Results

Table 1 shows the numbers of addresses, households and participants relevant to this analysis who took part in the Health Surveys for England in 1994, 1998 and 2003. Seventy five per cent of households (27,143-36,350) approached by the Health Survey for England in those years participated. Ninety-four per cent of people aged 45 and older (23,941-25,570) living in those households completed a questionnaire. Ninety six per cent of these (23,036-23,941) belonged to white ethnic groups (the ethnic group of people who did not complete a questionnaire is not known) and data on presence or absence of reported diagnosed cardiovascular disease were available for all but six of these.

**Table 1:** Participants in the Health Survey for England 1994, 1998 and 2003

	1994	1998	2003	All
Sampling units	720	720	720	

Addresses selected within sampling units	12,960	13,680	13,680	40,320

Eligible addresses*	11,515	12,250	12,036	35,801

Eligible households†	11,709	12,446	12,195	36,350

Co-operating households**	9,068	9,208	8,867	27,143

Adults aged 45+ living in co-operating households	8,257	8,688	8,625	25,570

Adults aged 45+ who completed individual questionnaire	7,751	8,181	8,009	23,941

White adults aged 45+ who completed individual questionnaire	7,492	7,908	7,636	23,036

White adults with data on cardiovascular disease prevalence	7,492	7,906	7,632	23,030

*ineligible addresses were those where no private households were found

† at some addresses has more than one household was resident there

**defined as households where at least one adult completed a questionnaire

Among white adults aged 45 and older, over the period studied, we found an increase in the proportions belonging to non-manual social classes (45.6% in 1994 to 53.1% in 2003); a decrease in the proportion who currently smoked (22.4% in 1994 to 19.0% in 2003); and an increase in the proportion with a body mass index greater than 30 kg m^-2 ^(18.4% in 1994 to 23.7% in 2003). We found a small increase in the prevalence of cardiovascular disease in white people aged 45 and older over the period studied (12.1% in 1994 to 13.7% in 2003). In each of the survey years, prevalence of cardiovascular disease increased with increasing age, lower social class, increasing body mass index and smoking (Table 2).

**Table 2:** Associations between social class, body mass index and smoking and prevalence of cardiovascular disease (CVD)

		1994		1998		2003	
		% CVD	Adjusted* OR (95% CI)	% CVD	Adjusted* OR (95% CI)	% CVD	Adjusted* OR (95% CI)

Registrar General's social class classification	I	8.5	1.00	8.2	1.00	9.4	1.00
	II	10.8	1.26 (0.86, 1.85)	10.6	1.35 (0.94, 1.93)	11.2	1.28 (0.92, 1.79)
	IIIa	11.7	1.17 (0.78, 1.75)	14.0	1.55 (1.06, 2.27)	14.6	1.55 (1.09, 2.20)
	IIIb	13.3	1.45 (1.00, 2.11)	13.8	1.69 (1.18, 2.41)	13.9	1.43 (1.03, 1.99)
	IV	14.5	1.53 (1.03, 2.27)	16.9	1.98 (1.37, 2.86)	16.2	1.76 (1.24, 2.49)
	IV	13.0	1.35 (0.87, 2.11)	19.0	2.40 (1.59, 3.63)	22.1	2.45 (1.64, 3.64)
							
BMI** (kg m^-2^)	<20	8.4	1.00	11.4	1.00	12.6	1.00
	20–24	9.3	1.32 (0.81, 2.16)	10.4	1.06 (0.67, 1.67)	9.7	0.72 (0.44, 1.20)
	25–29	13.2	1.84 (1.14, 2.98)	12.6	1.23 (0.79, 1.92)	12.2	0.84 (0.51, 1.37)
	30–34	13.3	1.91 (1.15, 3.16)	14.0	1.49 (0.94, 2.36)	14.6	1.08 (0.65, 1.79)
	35+	13.8	2.34 (1.32, 4.12)	15.7	2.07 (1.24, 3.46)	15.7	1.51 (0.88, 2.61)
							
Smoking	Never	9.2	1.00	10.4	1.00	10.9	1.00
	Ex-	15.6	1.52 (1.28, 1.82)	17.0	1.45 (1.24, 1.70)	17.1	1.36 (1.16, 1.59)
	Current	9.5	1.19 (0.95, 1.49)	10.9	1.27 (1.04, 1.56)	11.5	1.38 (1.12, 1.70)

*adjusted for age and sex **Body Mass Index

We had data on air pollution exposure for 93% of white adults aged 45 and older in 1994, 96% in 1998 and 99% in 2003. Table 3 shows summary statistics of outdoor air pollution exposure estimates at postcode sector level, for participating white adults aged 45 and older. For all pollutants, the variation across England became less marked between 1994 and 2003.

**Table 3:** Pollutant exposure at postcode sector level for white adults aged 45 and older

	1994			1998			2003		
	Median	Range	IQR*	Median	Range	IQR*	Median	Range	IQR*
PM_10_	19.6	12.5–36.1	3.7	18.0	12.6–27.0	2.9	16.3	11.0–22.6	2.6
NO_2_	27.3	4.1–73.0	17.8	35.6	7.3–66.7	12.7	23.1	6.1–54.6	10.9
SO_2_	9.5	1.0–41.1	8.3	6.2	0.2–39.3	6.1	4.2	0.5–14.4	2.8
O_3_	53.4	38.3–64.7	4.4	50.7	43.5–63.1	4.3	56.0	47.8–65.7	3.8

All units are μg m^-3^. 1998 and 2003 are average exposures over that year and the previous year. 1994 data are 1994 annual averages.

*Interquartile range

Average PM_10 _and SO_2 _concentrations fell over the period. NO_2 _concentrations increased between 1994 and 1998 and fell to a lower level in 2003. We saw the opposite pattern for O_3_.

**Figure 1:**
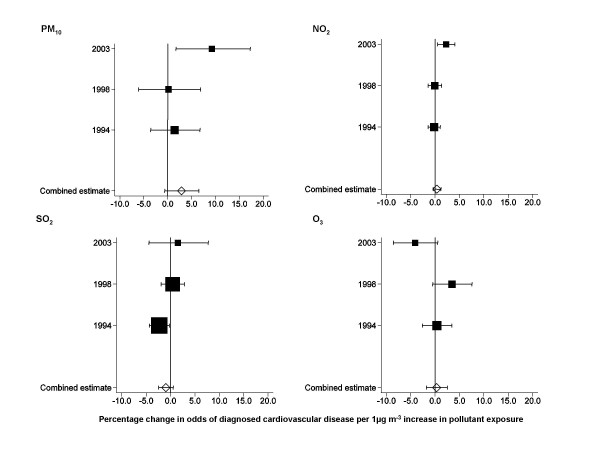
**Forest plots of results of multilevel logistic regression models of associations between pollutant exposure and diagnosed cardiovascular disease in men**. Horizontal lines correspond to 95% CI of estimated percentage change in odds of cardiovascular disease. Combined estimates are derived from fixed effects meta-analysis of year-specific estimates using inverse variance weighting. The size of the squares reflects the inverse variance of each year and, therefore, the weighting in the combined estimate. All estimates are adjusted for age (10 year age groups), social class of head of household (6 groups), body mass index (quartiles), cigarette smoking (never, ex-, current, and region of residence (8 groups), all as categorical variables.

**Figure 2:**
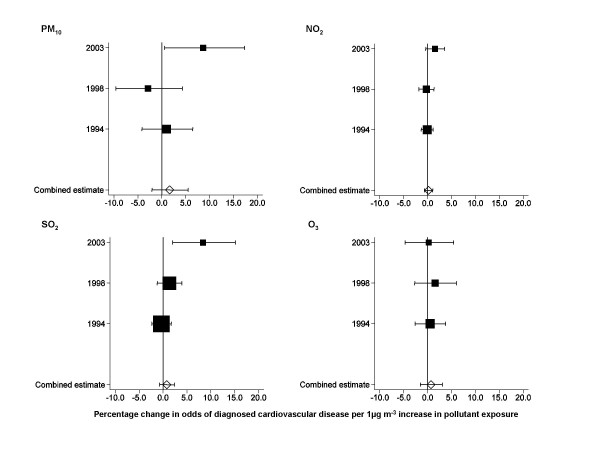
**Forest plots of results of multilevel logistic regression models of associations between pollutant exposure and diagnosed cardiovascular disease in women**. Horizontal lines correspond to 95% CI of estimated percentage change in odds of cardiovascular disease. Combined estimates are derived from fixed effects meta-analysis of year-specific estimates using inverse variance weighting. The size of the squares reflects the inverse variance of each year and, therefore, the weighting in the combined estimate. All estimates are adjusted for age (10 year age groups), social class of head of household (6 groups), body mass index (quartiles), cigarette smoking (never, ex-, current, and region of residence (8 groups), all as categorical variables.

Tables 4 and 5 provide crude and adjusted results of multilevel models estimating the associations between each of the four pollutants and prevalence of diagnosed cardiovascular disease for each survey year, and the results of the meta-analysis. Figures 1 and 2 summarise the adjusted estimates as forest plots. Our results are presented per 1 μg m^-3 ^increase in concentration. In 2003, a 1 μg m^-3 ^increase in concentration would have represented about 1/2 to 1/3 of the interquartile range of PM_10_, about 1/11 of the interquartile range of NO_2_, about 1/3 of the interquartile range of SO_2 _and about 1/4 of the interquartile range of O_3_.

**Table 4:** Associations between pollutant exposure and cardiovascular disease in men

		1994		1998		2003		Combined estimate*	
		n	% increase (95% CI)	n	% increase (95% CI)	n	% increase (95% CI)	n	% increase (95% CI)
PM_10_	Crude	3073	-1.79	3427	-1.32	3331	8.24		
			(-5.78, 2.36)		(-5.60, 3.15)		(2.95, 13.81)		
	Adjusted	2823	1.46	3034	0.16	2913	9.22	8770	2.88
			(-3.55, 6.73)		(-6.10, 6.84)		(1.72, 17.26)		(-0.64, 6.51)
NO_2_	Crude	3073	-0.54	3427	-0.18	3331	2.53		
			(-1.45, 0.37)		(-1.23, 0.88)		(1.25, 3.82)		
	Adjusted	2823	-0.21	3034	-0.08	2913	2.24	8770	0.37
			(-1.45, 1.04)		(-1.47, 1.33)		(0.47, 4.03)		(-0.45, 1.20)
SO_2_	Crude	3073	-1.27	3427	1.12	3343	5.09		
			(-2.83, 0.33)		(-0.75, 3.02)		(0.51, 9.88)		
	Adjusted	2823	-2.33	3034	0.42	2924	1.47	8781	-0.97
			(-4.35, -0.27)		(-1.94, 2.83)		(-4.43, 7.74)		(-2.47, 0.54)
O_3_	Crude	3042	0.85	3405	0.62	3313	-2.33		
			(-1.30, 3.03)		(-2.04, 3.35)		(-5.30, 0.74)		
	Adjusted	2785	0.35	3012	3.45	2903	-4.12	8700	0.33
			(-2.64, 3.43)		(-0.50, 7.56)		(-8.55, 0.51)		(-1.79, 2.49)

Estimates are % increase in odds of cardiovascular disease with 1 μg m^-3 ^increase in pollutant concentration

Adjusted estimates are adjusted for age (10 year age groups), social class of head of household (6 groups), body mass index (quartiles), cigarette smoking (never, ex-, current, and region of residence (8 groups), all as categorical variables

*Combined estimate is derived from a fixed effects meta-analysis of year-specific estimates using inverse variance weighting.

**Table 5:** Associations between pollutant exposure and cardiovascular disease in women

		1994		1998		2003		Combined estimate*	
		n	% increase (95% CI)	n	% increase (95% CI)	n	% increase (95% CI)	n	% increase (95% CI)
PM_10_	Crude	3904	1.63	4202	-0.89	4182	6.47		
			(-2.52, 5.96)		(-5.26, 3.67)		(1.09, 12.15)		
	Adjusted	3385	1.02	3596	-2.91	3469	8.63	10440	1.61
			(-4.14, 6.47)		(-9.62, 4.30)		(0.58, 17.32)		(-2.10, 5.45)
NO_2_	Crude	3904	0.12	4202	0.34	4182	1.78		
			(-0.79, 1.04)		(-0.75, 1.44)		(0.44, 3.13)		
	Adjusted	3385	-0.10	3596	-0.27	3469	1.55	10440	0.18
			(-1.35, 1.16)		(-1.83, 1.31)		(-0.39, 3.53)		(-0.69, 1.07)
SO_2_	Crude	3904	0.30	4202	2.01	4197	10.46		
			(-1.24, 1.86)		(0.16, 3.90)		(5.72, 15.40)		
	Adjusted	3385	-0.34	3596	1.32	3482	8.35	10463	0.80
			(2.34, 1.70)		(-1.22, 3.92)		(1.98, 15.12)		(-0.73, 2.36)
O_3_	Crude	3851	-0.60	4169	-0.04	4153	-3.21		
			(-2.76, 1.60)		(-2.79, 2.79)		(-6.20, -0.12)		
	Adjusted	3333	0.51	3568	1.55	3453	0.23	10354	0.74
			(-2.64, 3.75)		(-2.72, 6.00)		(-4.70, 5.43)		(-1.53, 3.07)

Estimates are % increase in odds of cardiovascular disease with 1 μg m^-3 ^increase in pollutant concentration

Adjusted estimates are adjusted for age (10 year age groups), social class of head of household (6 groups), body mass index (quartiles), cigarette smoking (never, ex-, current, and region of residence (8 groups), all as categorical variables

*Combined estimate is derived from a fixed effects meta-analysis of year-specific estimates using inverse variance weighting.

The combined estimates per 1 μg m^-3 ^increase in concentration of gaseous pollutants were small and all confidence intervals were consistent with no association. For exposure to PM_10_, the combined estimate suggested a 2.9% increase in odds of cardiovascular disease in men and 1.6% increase in women per 1 μg m^-3 ^increase in PM_10_, although the 95% confidence interval was consistent with no association in both sexes.

The year-specific analyses showed positive associations in 2003 between odds of cardiovascular disease in both men and women and exposure to PM_10 _with no similar pattern in 1994 or 1998. We also found, in 2003, an increase in odds of cardiovascular disease with increasing SO_2 _in women, but no similar pattern in men, and an increase in odds of cardiovascular disease with increasing NO_2 _in men, but no similar pattern in women. In 1994, the combined estimate for SO_2 _in men suggested a negative association between exposure and cardiovascular disease.

## Discussion

Our pooled analysis of data from three years of the Health Survey for England, which included over 19,000 white men and women aged 45 and older, demonstrates the associations between chronic exposure to air pollutants and prevalence of self-reported doctor-diagnosed cardiovascular disease. We found no consistent associations between chronic exposure to gaseous air pollutants and cardiovascular disease. We found a 2.9% increase in odds of cardiovascular disease in men and 1.6% increase in women per 1 μg m^-3 ^increase in PM_10_, although the 95% confidence intervals were consistent with no association.

Keys strengths of our study were that it was large and nationally representative with a high level of participation, and allowed us to examine effects over a wide range of exposures and to incorporate rich data on potential confounders. Using multilevel regression models is likely to have improved the accuracy and precision of our estimates, allowing for the possibility that risk of cardiovascular disease in people living near to each other may be more similar than in people living in other postcode sectors, because they may share other risk factors due to shared geography. Our estimates are unlikely to be affected by clustering of cardiovascular disease within households because we estimated associations in men and women separately.

Response was high to each level of request to participate in the Health Survey for England: 75% of households approached participated; 91% of adults living in participating households completed a questionnaire; and we had air pollution data available on more than 93% of the people included in our analysis. However, we had very limited information available about households who did not participate and adults who did not complete a questionnaire with which to assess the effect of non-response on our results.

All methods of estimating individual exposure to air pollutants will misclassify individual exposure to a degree, because measuring actual exposure is impractical, especially in large scale studies. Most commonly, researchers have used monitoring station data to assign exposure to all individuals living in large areas, which are likely to reflect individual exposure poorly. Other researchers have used population density[2] or estimated traffic exposure to improve on these [9]. Our method of estimating individual exposure to outdoor air pollutants may be more accurate because it takes account many influences on air pollution levels. We estimated exposure using comprehensive emission inventories combined with air dispersion models taking into account meteorological information [8]. The emission inventories were constructed using information on any influence on air pollution levels likely to have a measurable effect (including population density, emissions from roads from a combination of traffic activity data, type of vehicle, railways, airports, industry and domestic heating) [10], and enabled us to account directly for dispersion and processes in the atmosphere, providing added value over monitoring station data or simple land use methods examining only a handful of influences on air pollution levels. We used a more empirical approach to model ozone concentrations [11]. A comparison of modelled estimates with independent data from local authority monitoring sites showed good correlation for NO_2 _(r^2 ^= 0.85)[12] and O_3 _(r^2 ^= 0.81–0.89),[13] and reasonable correlation for PM_10 _(r^2 ^= 0.32–0.34)[12].

For people living very close to main roads, proximity to the road may be a better determinant of exposure than modelled levels. People living less than 50 metres from a main road have an increased prevalence of coronary artery calcification, a predictor of coronary events [14]. While we did not incorporate data on distance of residence from main road this is unlikely to have influenced our results: people living so close to main roads for this to be an important influence on health make up a very small proportion of the population and most of the fall in levels in particulate matter occurs within the first 10 metres from the road [15].

We did not have measures of fine particulates (particulate matter less than 2.5 μm in diameter (PM_2.5_) which are thought to be the most toxic. There are very few PM_2.5 _measurements in the UK on which to assess exposure. Studies comparing levels of PM_10 _and PM_2.5 _in the UK have found them to be strongly associated: Harrison and colleagues found strong correlations between hourly PM_10 _and PM_2.5 _measurements at five monitoring sites in the UK (r^2 ^of between 0.59 and 0.94) [16]. The PM_2.5_/PM_10 _ratio varied from 0.63 to 0.73. Moreover, some of the most influential studies have found relationships between cardiovascular mortality and PM_10 _that are similar to those with PM_2.5_[1]

We believe that participant-reported doctor-diagnosed angina, heart attack or stroke is a valid measure of cardiovascular disease. It was related to known risk factors as we would expect. In the British Regional Heart Study, study participants' reports of stroke and heart attack showed high levels of agreement with reports of stroke and heart attack from primary care records [17].

We are unable to explain why the estimates of association between cardiovascular disease and some of the measures of air pollution were most marked in 2003. We found no differences in the characteristics of the participants or their exposure (for example, regional effects) that could explain it. We have considered that there could have been something unusual about particle constituents in 2003 that could have caused this, but the absence of regional effects make this unlikely. We conclude that the striking findings in 2003 may be due to chance variation. If we had presented only the 2003 data, our conclusions would have been very different, suggesting a 90% increase in prevalence of cardiovascular disease per 10 μg m^-3 ^increase in PM_10_; in other words, large effects that would have been considered newsworthy. This highlights the importance of replicating findings in more than one population before drawing firm conclusions, and the pitfalls of selective publication. Publication bias has been demonstrated in studies of short term associations between air pollution and health effects [18].

The UK Committee on the Medical Effects of Air Pollution has estimated that the excess risk of cardiopulmonary death associated with a 10 μg m^-3 ^increase in PM_2.5 _is 9% (95% CI 3% to 16%) [19]. Although we examined prevalence of cardiovascular disease and not cardiopulmonary death, the overall effect size we observed is three to six times larger: in England, about two thirds of PM_10 _is made up PM_2.5 _and this varies little across the country; an increased odds of cardiovascular disease of 2.9% in men and 1.6% in women per 1 μg m^-3 ^increase in PM_10 _equates to an increase in odds of cardiovascular disease of 53% in men and 27% in women per 10 μg m^-3 ^increase in PM_2.5_. The United States study of cardiovascular events in women[4] found an even larger increase in risk of cardiovascular death per 10 μg m^-3 ^increase in PM_2.5 _(~80%), although that study's findings on risk of cardiovascular events were of a similar order to ours (20% increase per 10 μg m^-3 ^increase in PM_2.5_).

## Conclusion

Our estimates provide some support for other studies of the effects of PM_10 _on cardiovascular disease. The combined estimates of all the years' data showed a weakly positive association between PM_10 _and cardiovascular disease that was more consistent than for gaseous pollutants and was present in both men and women. Variation in year-specific estimates highlights the importance of replicating findings in more than one population. We found no consistent associations between chronic exposure to gaseous air pollutants and diagnosed cardiovascular disease.

## List of abbreviations

CVD: cardiovascular disease; PM_10_: particulate matter less than 10 microns in diameter; PM_2.5_: particulate matter less than 2.5 microns in diameter; NO_2_: nitrogen dioxide; SO_2_: sulphur dioxide; O_3_: ozone; μg m^-3^: microgrammes per cubic meter; km^2^: square kilometre; kg m^-2^: kilogramme per square metre; IQR: interquartile range; OR: odds ratio; CI: confidence interval.

## Competing interests

The authors declare that they have no competing interests.

## Authors' contributions

LF designed the study, co-led the analysis and drafted the paper. MP contributed to design, performed the statistical analyses, and contributed to drafting the paper. AR contributed to design, co-led statistical analysis, and contributed to drafting the paper

DC contributed to design and conception of the study, the analysis and interpretation, and critically reviewed the paper. DS contributed to design and conception of the study, the analysis and interpretation of the data, and critically reviewed the paper. TB contributed to design of the study, did analyses of air pollution data, and critically reviewed the paper

JS contributed to design of the study, did analyses of air pollution data, and critically reviewed the paper. PW contributed to design and conception of the study, contributed to interpretation of the data, and critically reviewed the paper. HRA contributed to design and conception of the study, contributed to interpretation of the data, and critically reviewed the paper. All authors read and approved the final manuscript.

## Supplementary Material

Additional file 1 **Models for estimating exposure to air pollutants**. Further details of modelling exposure to air pollutants: emission inventories, modelling methods and validation. Click here for file
