# Supplementary material for: Chronic exposure to outdoor air pollution and diagnosed cardiovascular disease: meta-analysis of three large cross-sectional surveys
Source: Environ Health. 2009 Jul 13;8:30. doi: 10.1186/1476-069X-8-30 (PMC2716315; doi:10.1186/1476-069X-8-30)
Supplement: Additional file 1 — Models for estimating exposure to air pollutants. Further details of modelling exposure to air pollutants: emission inventories, modelling methods and validation. [file 1476-069X-8-30-S1.doc]

### Additional file 1: Models for estimating exposure to air pollutants

### Emission inventory

Emission estimates were provided by the UK National Atmospheric Emission Inventory.^1^ Funded by the Government, the National Atmospheric Emission Inventory provides detailed information on a air pollutant emissions as part of national air quality monitoring and assessment systems. It quantifies emissions from statutorily regulated point sources (e.g. industrial sites) which are reported by the Environment Agency (a public body accountable to parliament). Emissions from area sources (e.g. roads, railways) are typically derived from a combination of activity statistics (such as fuel consumption) and known emission factors (for example, emission of a given pollutant per tonne of a given fuel burnt or kilometre travelled). The emissions are estimated for a wide variety of activities and then aggregated into sectors such as domestic heating, road traffic emissions and industrial emissions. The UK total emission estimates for each activity are then distributed across 1 km x 1 km squares covering the whole of the UK. The spatial pattern of emissions are derived from information based on population or employment statistics for a number of activities. Emissions from road traffic on major roads have been estimated in great detail from a combination of traffic activity data (daily flows for different vehicle types on each of more than 19,000 major road links), vehicle fleet characteristics (age, relevant emission standard) and emission factors (emissions per km per vehicle).

### Oxides of nitrogen

*Introduction*

Concentrations of NO_x_ and NO_2_ were estimated using the Pollution Climate Mapping model.^2;3;4^

### *Modelling NO_x_ concentrations*

Annual mean NO_x_ concentrations at background locations were calculated by summing the estimated concentrations for the following components:

- Distant sources (characterised by the rural background concentration, interpolated from measurements at rural sites)
- Point sources (calculated using an air dispersion model)
- Local area sources (calculated using a kernel based air dispersion model)

The area source model has been calibrated using data from the national automatic monitoring networks for the relevant year.

### *Modelling NO_2_ concentrations*

To estimate NO_2_ concentrations, modelled NO_x_ concentrations derived from the approach outlined above are converted to NO_2_ using an oxidant partitioning model which describes the complex inter-relationships of NO, NO_2_ and ozone as a set of chemically coupled species.^5^ This approach provides additional insights into the factors controlling ambient levels of NO_2_ (including the emission of primary NO_2_), and how they may vary with NO_x_ concentration.

*Verification of model results*

We compared modelled estimates of NO_x_ and NO_2_ concentrations with those from fixed monitoring sites across the UK (both automatic national network sites and a number of other verification monitoring sites that are not included in the national network). Table III.1 shows the results of comparisons between modelled and measured concentrations for 2003, demonstrating the high level of agreement.

**Table III.1. Comparison between modelled and measured annual mean NO_x_ and NO_2_** concentrations at background sites

|  |  | Number of sites | Mean of measurements at monitoring sites (μg m^-3^, as NO_2_) | Mean of model estimates  (μg m^-3^, as NO_2_) | r^2^ |
| --- | --- | --- | --- | --- | --- |
| NO_x_ | National network | 64 | 55.8 | 53.7 | 0.79 |
|  | Verification sites | 19 | 54.5 | 55.7 | 0.87 |
| NO_2_ | National network | 64 | 31.4 | 31.6 | 0.83 |
|  | Verification sites | 19 | 30.3 | 31.9 | 0.87 |

**Particulate matter**

*Introduction*

PM_10_ concentrations were estimated using the Pollution Climate Mapping model.^3;6^

### *Modelling PM_10_ concentrations*

Annual mean PM_10_ concentrations at background locations were calculated by summing the estimated concentrations for the following components:

- Secondary inorganic aerosol (derived by scaling measurements of PM, SO_4_, NO_3_)
- Point sources of primary particles (modelled using an air dispersion model and UK national emissions estimates from the National Atmospheric Emission Inventory.
- Area sources of primary particles (modelled using a dispersion kernel, which is derived using an air dispersion model and emissions estimates from the National Atmospheric Emission Inventory)
- Residual (assumed to be a constant value)

The area source model was calibrated using data from the national automatic monitoring networks for the relevant year.

*Verification of model results*

We compared modelled estimates of PM_10_ concentrations with those from fixed monitoring sites across the UK (both automatic national network sites and other verification monitoring site). Table III.2 shows the results of comparisons between modelled and measured concentrations for 2003, demonstrating a reasonable level of agreement.

PM concentrations are not generally characterised as well within a dispersion model as a gaseous pollutant such as NO_x_ because airborne PM is a complex mixture for pollutants for which the sources are not as well characterised.

**Table III.2. Comparison between modelled and measured annual mean concentrations of PM_10_** at background sites (using Tapered Element Oscillating Microbalance)

|  | Number of sites | Mean of measurements at monitoring sites  (μg m^-3^) | Mean of model estimates  (μg m^-3^) | r^2^ |
| --- | --- | --- | --- | --- |
| National network | 47 | 19.6 | 18.0 | 0.25 |
| Verification sites | 20 | 20.0 | 18.9 | 0.37 |

### Sulphur dioxide

*Introduction*

SO_2_ concentration estimates were estimated using the Pollution Climate Mapping model.^3^

### *Modelling SO_2_ concentrations*

Annual mean SO_2_ concentrations at background locations were calculated by summing the estimated concentrations for the following components:

- Point sources (calculated using an air dispersion model)
- Local area sources (calculated using a kernel based air dispersion model)

Point sources (such as power stations and refineries) are the dominant contributor to ground level concentrations across most of the UK.

*Verification of model results*

As for NO_x_ and PM_10_, we compared modelled estimates of SO_2_ concentrations with those from fixed monitoring sites across the UK. Table III.3 shows the results of comparisons between modelled and measured concentrations for 2003, demonstrating a reasonable level of agreement.

**Table III.3. Comparison between modelled and measured annual mean concentrations of SO_2_** at background sites

|  | Number of sites | Mean of measurements at monitoring sites  (μg m^-3^) | Mean of model estimates  (μg m^-3^) | r^2^ |
| --- | --- | --- | --- | --- |
| National network | 108 | 5.5 | 4.4 | 0.45 |
| Verification sites | 43 | 6.7 | 6.3 | 0.56 |

**Ozone**

*Introduction*

The empirical mapping methods used to calculate the maps of annual mean ozone concentration have been described by Stedman and Kent^7^ as a further development of the methods presented by Coyle at al.^8^

*Mapping ozone concentrations*

The maps of annual mean concentration were calculated by interpolating monitoring data from rural monitoring sites for the well-mixed period in the afternoon (12:00 to 18:00). Two corrections were then applied: to correct for altitude and for the effect of urban environments. The altitude correction was applied to take account of the effects of topography on ozone levels.^8^ Topographic effects are important for some ozone metrics, such as the annual mean, because of the disconnection of a shallow boundary layer from air aloft during the night at lowland locations. Surface ozone concentrations are lower at night in these locations due to a combination of dry deposition and titration with NO emissions. This effect is much less marked at higher altitudes and at coastal locations, where wind is generally stronger and a shallow boundary layer does not form. An urban decrement (the difference between the ozone concentration in an urban area and the regional rural value) was then calculated using the oxidant partitioning model of Jenkin.^5^ Maps of regional oxidant were calculated as the sum of altitude corrected ozone and rural NO_2_ as interpolated from measurements at rural sites. The partitioning of oxidant between ozone and NO_2_ was then calculated as a function of local NO_x_ concentrations, as modelled using the approach described above. Maps of annual mean ozone concentration were calculated for all of the 1 km x 1 km squares in the UK by subtracting this urban decrement from the estimates of rural concentrations.

*Verification of model results*

We compared modelled ozone estimates and measurements from fixed monitoring sites in the national network across the UK. Table III.4 shows the results of comparisons between modelled and measured concentrations of ozone for 2003, demonstrating good agreement between them.

**Table III.**4. Comparison between modelled and measured annual mean concentrations of ozone at background sites

|  | Number of sites | Mean of measurements at monitoring sites (μg m^-3^) | Mean of model estimates (μg m^-3^) | r^2^ |
| --- | --- | --- | --- | --- |
| National network | 80 | 45.2 | 44.8 | 0.73 |

Results of analysis of between-postcode and within postcode sector variance in air pollutant concentrations

Table III.5 shows variation in pollutant levels between 4178 postcode sectors in England, Scotland and Wales 1991-1994.

Table 5. Variability of air pollution measures between and within postcode sector

|  | % of variance explained by between-sector variation | Intraclass correlation within sector |
| --- | --- | --- |
| PM_10_ | 97.7 | 0.941 |
| NO_2_ | 99.7 | 0.993 |
| SO_2_ | 98.7 | 0.968 |
| O_3_ | 98.3 | 0.955 |

**References**

(1) Dore CJ, Watterson JD, Murrells TP, Passant NR, Hobson MM, Baggott SL, Thistlethwaite G, Goodwin JWL, King KR, Adams M, Downes MK, Walker C, Coleman PJ, Stewart RA, Wagner A, Sturman J, Conolly, C, Lawrence, H, Li Y, Jackson J, Bush T, Grice S, Brophy, N. UK emissions of air pollutants 1970 to 1994. AEAT/ENV/R/2359. 2007. Didcot, Oxfordshire, National Atmospheric Emissions Inventory, AEA Technology, National Environmental Technology Centre.

(2) Stedman JR, Vincent KJ, Campbell GW, Goodwin JWL, Downing CEH. New high resolution maps of estimated background ambient NOx and NO2 concentrations in the U.K. Atmospheric Environment 1997; 31(21):3591-3602.

(3) Stedman JR, Bush TJ, Vincent KJ, Kent AJ, Grice S, Abbott J. UK air quality modelling for annual reporting 2003 on ambient air quality assessment under Council Directives 96/62/EC, 1999/30/EC and 2000/69/EC. Report AEAT/ENV/R/1790. 2005. Didcot, Oxfordshire, AEA Technology, National Environmental Technology Centre.

(4) Stedman JR, Goodwin JWL, King K, Murrells TP, Bush TJ. An empirical model for predicting urban roadside nitrogen dioxide concentrations in the UK. Atmospheric Environment 2001; 35(8):1451-1463.

(5) Jenkin ME. Analysis of sources and partitioning of oxidant in the UK--Part 1: the NOX-dependence of annual mean concentrations of nitrogen dioxide and ozone. Atmospheric Environment 2004; 38(30):5117-5129.

(6) Stedman JR, Kent AJ, Grice S, Bush TJ, Derwent RG. A consistent method for modelling PM10 and PM2.5 concentrations across the United Kingdom in 2004 for air quality assessment. Atmospheric Environment 2007; 41(1):161-172.

(7) Stedman JR, Kent AJ. An analysis of the spatial patterns of health related surface ozone metrics across the UK in 1995, 2003 and 2005. Atmospheric Environment 2008.

(8) Coyle M, Smith RI, Stedman JR, Weston KJ, Fowler D. Quantifying the spatial distribution of surface ozone concentration in the UK. Atmospheric Environment 2002; 36(6):1013-1024.
